# Anesthesia efficacy of bupivacaine in pregnant participants with breech presentation receiving external cephalic version: A protocol of systematic review of randomized controlled trials

**DOI:** 10.1097/MD.0000000000020786

**Published:** 2020-06-19

**Authors:** Xin-hua Mu, Hai-Xia Shi, Ran An

**Affiliations:** a Department of Anaesthesiology, The Affiliated Hospital of Inner Mongolia Medical University; b Department of Emergency, The Second Affiliated Hospital of Inner Mongolia Medical University, Hohhot, China.

**Keywords:** breech presentation, bupivacaine, efficacy, external cephalic version, pain relief, safety

## Abstract

**Background:**

The objective of this study is to appraise the efficacy and safety of bupivacaine in pregnant participants with breech presentation (BP) receiving external cephalic version (ECV).

**Methods:**

The following electronic databases will be searched from the origin to the January 31, 2020: PUBMED, EMBASE, Cochrane Library, CINAHL, ACMD, PsycINFO, Scopus, OpenGrey, and China National Knowledge Infrastructure. No language and publication time limitations will be applied to all of them. Randomized controlled trials comparing bupivacaine to other interventions for pain relief in pregnant participants with BP undergoing ECV will be included in this study. Two authors will employ the selection of searched records, extraction of essential data from included RCTs, and risk of bias assessment for each eligible trail independently and respectively. Any doubts between 2 authors will be figured out by a third author through discussion. The risk of bias assessment will be judged using Cochrane risk of bias tool. The data pooling and analysis will be performed using RevMan 5.3 software.

**Results:**

This study will summarize the up-to-date high-quality evidence and will synthesis the outcome data from that evidence to explore the efficacy and safety of bupivacaine for pain relief in pregnant participants with BP undergoing ECV.

**Conclusion:**

The findings of this study may present important guidance for patients, clinical practice, as well as health-policy makers regarding the utilization of bupivacaine for pain relief in pregnant participants with BP receiving ECV.

**Systematic review registration:**

PROSPERO CRD42020164409.

## 1 Introduction

Breech presentation (BP) occurs in 3% to 4% of all deliveries in pregnant women, which often involves cesarean delivery.^[1–4]^ It is associated with an increased risk for congenital malformations and mild deformations, as well as maternal and fetal morbidity.^[5–7]^ To avoid incidence of these risk factors and cesarean delivery, it is very necessary to manage BP in pregnant females.^[8–11]^

Previous studies suggested external cephalic version (ECV) can help manage BP disorder,^[12–15]^ and all participants with BP receiving ECV experience very severe pain intensity,^[13–18]^ with mean scores of 4.6 to 8.5 out of 10, as examined by visual analog scale.^[7]^ Several clinical studies reported the efficacy and safety of bupivacaine for pain relief in pregnant participants with BP undergoing ECV.^[19–23]^ However, no systematic review exploring this issue exists. Thus, this study will systematically and comprehensively assess the efficacy and safety of bupivacaine for pain decrease in BP receiving ECV.

## 2 Methods

### 2.1 Dissemination and ethics

This study will be disseminated on a peer-reviewed journal or a relevant conference. No ethic approval document is required in this study because it will not employ any individual data.

### 2.2 Study registration

We have registered this study on PROSPERO (CRD42020164409). We have reported this study based on the guidelines of Cochrane Handbook for Systematic Reviews of Interventions and the Preferred Reporting Items for Systematic Reviews and Meta-Analysis Protocol statement.^[24]^

### 2.3 Inclusion criteria for study selection

#### 2.3.1 Types of studies

We will include all potential randomized controlled trials (RCTs) that appraise the efficacy and safety of bupivacaine in pregnant participants with BP receiving ECV. No language and publication status limitations will be applied.

#### 2.3.2 Types of participants

Inclusion criteria for study participants are pregnant adult females (18 years old or older) with BP undergoing ECV. We will not implement any restrictions in terms of country, race, and educational background.

#### 2.3.3 Types of interventions

In the experimental group, all participants received bupivacaine alone as their managements. However, we will exclude bupivacaine combined with any treatments.

In the control group, any anesthetic management could be utilized, but not any forms of bupivacaine.

#### 2.3.4 Type of outcome measurements

Primary outcome is pain intensity, as assessed by any pain scales reported in the trials.

Secondary outcomes are analgesic consumption, success rate of ECV, maternal satisfaction for ECV (as measured by any relevant tools reported in the trials), and adverse events.

### 2.4 Data sources

#### 2.4.1 Electronic databases search

The following electronic databases will be retrieved from their beginning up to the January 31, 2020: PUBMED, EMBASE, Cochrane Library, CINAHL, ACMD, PsycINFO, Scopus, OpenGrey, and China National Knowledge Infrastructure. We will not exert language and publication time limitations to all of them. The sample of search strategy of PUBMED is created in Table 1. We will also adapt similar search strategies to the other electronic databases.

**Table 1:**
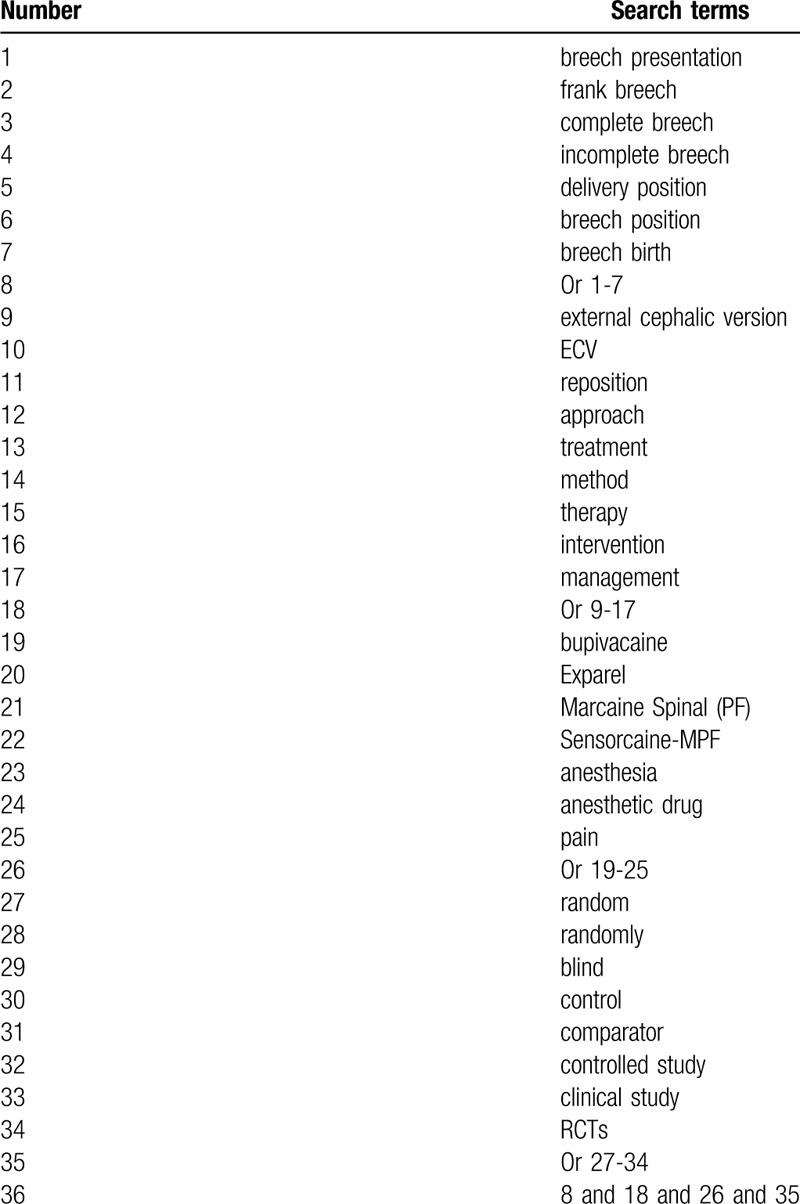
Search strategy for PUBMED database.

#### 2.4.2 Search for other resources

In addition to the above electronic databases, we will identify conference abstracts, clinical trials registry, and reference lists of all included trials for potential available studies.

### 2.5 Study selection and data management

#### 2.5.1 Study selection

The whole process of study selection consists of 2 stages. First, tiles/abstracts of all searched studies will be independently scanned by 2 experienced authors according to the eligibility criteria. All duplicates and irrelevant studies will be excluded. Second, full article of the remaining studies will be read carefully against all inclusion criteria. Any divisions between 2 authors will be worked out with the help of another experienced author through consultation or discussion. The process of study search and selection is shown in a flow diagram.

#### 2.5.2 Data extraction and management

Two experienced authors will independently extract the essential information from all eligible trials using predefined standardized data extraction form. Any deviations will be coped with another experienced author through consultation and a final decision will be made. The collected information includes study title, location, first author, publication year, participant characteristics (such as age, race, diagnostic criteria, and eligibility criteria), trial setting, trial methods, sample size, specifics of interventions and controls, outcomes, safety, follow-up information, results, findings, conflict of interest, and funding details. We will contact original authors by email if we find some missing or unclear information.

#### 2.5.3 Study quality assessment

Two experienced authors will independently appraise the study quality using Cochrane risk of bias tool. This tool consists of 7 domains, and each item is further rated into 3 levels: low risk of bias, unclear risk of bias, and high risk of bias. Any arguments between 2 authors will be resolved by another experienced author through discussion or consultation.

### 2.6 Statistical analysis

Statistical analysis will be performed using RevMan 5.3 software. Regarding the dichotomous data, such as success rate of ECV and incidence of adverse events, risk ratio or odds ratio and 95% confidence intervals (CIs) will be utilized. Regarding the continuous data, such as pain intensity, analgesic consumption, and maternal satisfaction for ECV, mean difference or standardized mean difference and 95% CIs will be used. The statistical heterogeneity across the eligible trials will be determined by *I*^2^ test. *I*^2^ ≤50% means acceptable heterogeneity, and we will employ a fixed-effects model for data pooling. Otherwise, *I*^2^ >50% reveals obvious heterogeneity, and we will implement a random-effects model for data synthesizing. If acceptable heterogeneity is identified across the sufficient included trials, meta-analysis will be conducted based on the similar study information, patient characteristics, specifics of interventions and controls, and outcome measurements. Otherwise, we will carry out subgroup analysis to detect possible factors that may cause obvious heterogeneity.

### 2.7 Additional analysis

#### 2.7.1 Reporting bias

If at least 10 eligible trials enter this study, we will detect reporting bias using Funnel plot and Egger regression test.^[25]^

#### 2.7.2 Subgroup analysis

When there is obvious heterogeneity among included trials, subgroup analysis will be carried out in accordance with the different study information, participant characteristics, study quality, intervention, comparators, and outcome measurements.

#### 2.7.3 Sensitivity analysis

Whenever necessary, we will perform sensitivity analysis to investigate the robustness and stability of study findings by removing low-quality studies.

#### 2.7.4 Grading the quality of evidence

The strength of each outcome will be assessed using Grading of Recommendations Assessment, Development and Evaluation (GRADE) method.^[26]^ Two experienced authors will independently assess the quality of evidence for each outcome. Any divergences between 2 authors will be solved by another experienced author via discussion.

## 3 Discussion

A numerous studies have reported that bupivacaine has been utilized for pain relief in pregnant participants with BP undergoing ECV. However, their findings are still inconsistent, and no systematic review has been addressed focusing on such issue. Thus, it is very necessary and crucial to make sure whether bupivacaine is a good option for pain relief in pregnant participants with BP undergoing ECV. This study aims to systematically and comprehensively explore the efficacy and safety of the utilization of bupivacaine for pain relief in pregnant participants with BP receiving ECV. The findings of this study may provide helpful evidence for both clinicians and future researches.

## Author contributions

**Conceptualization:** Xin-hua Mu, Ran An.

**Data curation:** Ran An.

**Formal analysis:** Xin-hua Mu, Ran An.

**Investigation:** Ran An.

**Methodology:** Xin-hua Mu, Hai-Xia Shi.

**Project administration:** Ran An.

**Resources:** Xin-hua Mu, Hai-Xia Shi.

**Software:** Xin-hua Mu, Hai-Xia Shi.

**Supervision:** Ran An.

**Validation:** Xin-hua Mu, Hai-Xia Shi, Ran An.

**Visualization:** Xin-hua Mu, Hai-Xia Shi, Ran An.

**Writing – original draft:** Xin-hua Mu, Hai-Xia Shi, Ran An.

**Writing – review & editing:** Xin-hua Mu, Hai-Xia Shi, Ran An.
